# Polymorphism in Adiponectin and Adiponectin Receptor Genes in Diabetes Mellitus Pathogenesis

**DOI:** 10.3390/pathophysiology29010008

**Published:** 2022-02-28

**Authors:** Iuliana Shramko, Elizaveta Ageeva, Eugene Krutikov, Konstantin Maliy, Irina Repinskaya, Iryna Fomochkina, Anatolii Kubishkin, Anna Gurtovaya, Cyrill Tarimov, Suman Shekhar

**Affiliations:** 1 Department of General and Clinical Pathophysiology, S. I. Georgievsky Medical Academy of the Federal State Autonomous Educational Institution of Higher Education, V. I. Vernadsky Crimean Federal University, 295000 Simferopol, Russia; kubyshkin_av@mail.ru (A.K.); kirito.k@yandex.ru (C.T.); 2 Department of Medical Biology, S. I. Georgievsky Medical Academy of the Federal State Autonomous Educational Institution of Higher Education, V. I. Vernadsky Crimean Federal University, 295000 Simferopol, Russia; ageevaeliz@rambler.ru (E.A.); shramko_6767@mail.ru (A.G.); office@ma.cfuv.ru (S.S.); 3 Department of Propaedeutics of Internal Medicine, S. I. Georgievsky Medical Academy of the Federal State Autonomous Educational Institution of Higher Education, V. I. Vernadsky Crimean Federal University, 295000 Simferopol, Russia; nephrostar@yandex.ru; 4 Department of Biochemistry, S. I. Georgievsky Medical Academy of the Federal State Autonomous Educational Institution of Higher Education, V. I. Vernadsky Crimean Federal University, 295000 Simferopol, Russia; kdmalykd@gmail.com; 5 Department of Internal Medicine, S. I. Georgievsky Medical Academy of the Federal State Autonomous Educational Institution of Higher Education, V. I. Vernadsky Crimean Federal University, 295000 Simferopol, Russia; repinskaya.irina@mail.ru; 6 Department of Basic and Clinical Pharmacology, S. I. Georgievsky Medical Academy of the Federal State Autonomous Educational Institution of Higher Education, V. I. Vernadsky Crimean Federal University; 295000 Simferopol, Russia; fomochkina_i@mail.ru

**Keywords:** gene polymorphism, adiponectin, adiponectin receptors, diabetes mellitus

## Abstract

The role played by hereditary factors in the development of diabetes mellitus type 2 (DM2) has not yet been fully established. Therefore, the purpose of our study was to investigate the prevalence of adiponectin and polymorphism in its gene receptors in connection with the primary symptoms of DM2 pathogenesis. Genomic DNA was isolated from the whole blood of 94 patients with an established diagnosis of DM2 using the phenol–chloroform method. Gene polymorphisms were determined using real-time polymerase chain reaction (PCR). The most common polymorphic variants in patients with DM2 were the genotypes AA (rs11061971) and GG (rs16928751) on the ADIPOR2 gene. A strong correlation was found between the rs16928751 polymorphism on the ADIPOR2 gene and increased body mass index (BMI). TG (rs2275737) ADIPOR1 gene genotype carriers were found to have the highest levels of glycosylated hemoglobin (HbA1), whereas TT (rs2275738) caused stable hyperglycemia. In addition, the rs16928751 ADIPOR2 gene polymorphism showed an association with the development of key mechanisms of DM2 in the Russian population, although a number of genomic searches failed to show any association of this gene with DM2. Unique gene variants associated with the risk of developing DM2 in the Crimean population were established.

## 1. Introduction

In the pathogenesis that leads to type 2 diabetes mellitus (DM2), both hereditary predisposition and obesity as the result of lifestyle and nutrition habits are considered key players. For this reason, investigations of polymorphism in candidate genes involved in DM2 pathogenesis are of great practical importance. It has been established that DM2 is characterized by polygenic development. The adiponectin-mediated pathway, among others, is associated with impaired glucose tolerance, insulin resistance (IR), and obesity [1]. Adiponectin is encoded by a specific gene (ADIPOQ) and synthesized by white adipose tissue cells. A low concentration of adiponectin in the blood, typical for DM2 patients, is associated with a decrease in lipid oxidation, increased triglyceride concentrations, and compromised glucose consumption in peripheral tissues [2].

Adiponectin cannot exert its biological functions acting alone; instead, the protein hormone acts through binding with its receptors, ADIPOR1 and ADIPOR2 [3]. The expression of both genes is reduced in skeletal muscles in DM2 patients [4]. ADIPOR1 is associated with body weight gain and a decrease in insulin sensitivity, whereas ADIPOR2 is associated with a drop in both triglyceride concentrations and the level of lipid oxidation. In addition, it has been linked with a high risk of cardiovascular events. Therefore, it is crucial to gain an understanding, not only of the genetics of adiponectin, but also of ADIPOR1 and ADIPOR2, since heredity plays a key role in the pathogenesis of diabetes and other metabolic diseases associated with adiponectin [5]. 

A high number of single nucleotide polymorphisms (SNP) (e.g., rs2241766, rs1501299, rs266729, rs17366743, rs17300539, rs182052, rs822396, rs17846866, rs3774261, and rs822393) in the adiponectin gene have been found to be significantly associated with the pathogenesis of diabetes [6]. Three of the common SNP of ADIPOQ (rs266729, rs2241766, and rs1501299) have been the most widely studied. However, findings from previous studies of these SNP’s are inconsistent and inconclusive. Among the aforementioned mutational variants, SNP rs2241766 has been significantly linked to all three forms of the disease. Polymorphism rs1501299 may play a protective role in coronary artery disease (CAD) in diabetic patients. The rs2241766 polymorphism has been found to be associated with a significant increase in CAD risk in Caucasians [7]. 

In studies carried out in the Finnish population, three markers of the ADIPOR1 gene (rs10920534, rs12045862, and rs7539542) were found to be associated with body weight gain and a decrease in insulin sensitivity. The ADIPOR1 gene markers T (−102) and A5843G demonstrated a significant association with DM2 in US Caucasians. Additionally, the polymorphic marker T (−102) G was found to have an irregular coupling with the polymorphic marker T (−106) C [8].

Some variants of ADIPOR2 displayed an association with IR, as well as a decrease in both the concentration of triglycerides and the levels of lipid oxidation. In the Finnish population, the polymorphic marker G795A (rs16928751) of the ADIPOR2 gene was associated with the risk of development of cardiovascular disease in individuals with impaired glucose tolerance. However, studies of the association between ADIPOR2 gene variants and DM2 in several European populations have shown contradictory results [9]. 

To select particular polymorphisms for the research, we used data from meta-analyses [10,11,12,13] that demonstrated a high degree of association with DM2 for ADIPOQ + 45 T/G (rs2241766) and G276T (rs1501299). Additionally, we took into account that the ADIPOR2 + 795 G/A (rs16928751) variant was associated with higher plasma adiponectin levels and decreased fasting triglyceride, VLDL triglyceride, and VLDL cholesterol levels, all of which are significant components of MS (metabolic syndrome) and DM2 pathogenesis [14]. Besides that, the polymorphic markers rs2241766 (ADIPOQ), rs22753738 (ADIPOR1), and rs11061971 and rs16928751 (both in ADIPOR2), have been implicated in susceptibility to DM2 in the Russian population [15]. Concurrently, it has been reported that both ADIPOR 1 rs2275738 and rs2275737 have been associated with a risk for DM2 among an isolated Amish population, which may be an indicator of a homozygotic insulin-resistance group [16]. Hence, recent meta-analyses have shown a correlation between the expression of adiponectin receptor genes and insulin sensitivity in humans, the research data concerning the adiponectin receptor gene’s polymorphisms responsible for IR and the development of DM2 are both sparse and contradictory. Therefore, the purpose of our study was to investigate the prevalence of the adiponectin genes and their receptor polymorphisms in connection with the main clinical manifestations of DM2 pathogenesis.

## 2. Materials and Methods

### 2.1. Patient Selection

A continuous, single-center, case-control search study, performed simultaneously on samples of patients with DM2 and healthy residents of the Republic of Crimea was carried out. Two hundred and seven patients (107 females and 100 males) treated at Semashko Republican Hospital, Simferopol and 100 healthy individuals (59 females and 41 males) as a control group were involved in the study (Table 1). The study was conducted according to the guidelines of the Declaration of Helsinki 1975 (revised in 2013) and approved by the V.I.Vernadsky Crimean Federal University Ethics Committee (Protocol No. 8 from 17 January 2018). Informed consent was obtained from all subjects involved in the study.

### 2.2. Inclusion and Exclusion Criteria in the Study 

#### 2.2.1. Inclusion Criteria for Patients

- Male or female patients aged ≥52.0 to ≤70.0 years.
- Patients with a verified diagnosis of DM2 (target glycated hemoglobin (HbA1c) level < 7.0%; target fasting plasma glucose level < 7.0 mmol/L (2 h after meals, <9.0 mmol/L); and a glomerular filtration rate according to the Chronic Kidney Disease Epidemiology Collaboration (CKD–EPI) of 80 mL/min/1.73 m^2^).
- BMI of more than 30 kg/m^2^.
- Willingness to voluntarily participate in the study and able to sign an informed consent form.

#### 2.2.2. Exclusion Criteria for Patients

- Male or female patients aged ≤52.0 years or ≥70.0 years.
- Unstable DM2 (target HbA1c level > 7.0%; target fasting plasma glucose level > 7.0 mmol/L (2 h after meals, >9.0 mmol/L); a glomerular filtration rate according to Chronic Kidney Disease Epidemiology Collaboration (CKD–EPI) of <80 mL/min/1.73 m^2^).
- Chronic renal disease, heart failure, liver dysfunction, or malignant tumor.
- Inability or unwillingness to participate in the study or to sign an informed consent form.

#### 2.2.3. Inclusion Criteria for Control Subjects

- Normoglycemic men or women with no past history of either glucose intolerance or a family history of diabetes.
- Aged ≥52.0 to ≤70.0 years.
- HbA1c level of <6.4%, or a normal oral glucose tolerance test (OGTT).
- Individuals with BMI of less than 30 kg/m^2^.
- Willingness to voluntarily participate in the study and able to sign an informed consent form.

We have studied the polymorphisms of the ADIPOQ gene + 45 T/G (rs2241766) and G276T (rs1501299), the polymorphisms of the ADIPOR1 gene A/T (rs2275737) and C/T (rs2275738), and the polymorphisms of the ADIPOR2 genes + 219 A/T (rs11061971) and +795 G/A (rs16928751). 

### 2.3. Biochemical Measurements

Fasting levels of cholesterol and glucose were measured using standard methods. HbA1c was measured using ion-exchange high performance liquid chromatography (normal reference range: 4.1–6.4%). 

### 2.4. Physical Examination 

Body mass index (BMI), which is used to diagnose overweight and obesity, as well as to assess its degree, is calculated as follows: body weight (m) in kilograms divided by the square of height (h) in meters and reported as kg/m^2^ (WHO International Obesity Group (IOTF WHO), 1997).

Arterial hypertension is defined as diastolic blood pressure above 90 mmHg and/or systolic blood pressure above 140 mmHg.

### 2.5. PCR Technique

Genomic DNA was isolated from the whole blood of patients using the phenol–chloroform method. Real-time polymerase chain reaction (PCR) performed on a Bio-Rad CFX96 thermal cycler was used to determine gene polymorphisms. The reaction mixture contained 70 mM Tris HCl (pH 8.8), 16.6 mM ammonium sulfate, 250 nM fluorescent probes, 1.5 units of Taq DNA polymerase, oligonucleotide primers of Synthol, and 50–100 ng of genomic DNA.

Amplification was processed at 95 °C for 2 min, primer annealing + 45 T/G (rs2241766) at 65 °C, +276 G/T (rs1501299), +219 A/T (rs11061971), and +795 G/A (rs16928751) at 58 °C, −102 T/G (rs2275737) and −106 T/S (rs2275738) at 59 °C, for 40 cycles. The fluorescent dyes used in the probes were FAM (carboxyfluorescein) and HEX (hexachlorofluorescein); the fluorescence quencher was BHQ–1.

The study was performed in the Center for Collective Use of Scientific Equipment (Molecular Biology) of the S. I. Georgievsky Medical Academy (structural division) of the V. I. Vernadsky CFU. 

### 2.6. Statistical Analysis

Data obtained in this study were analyzed using the Statistica 8.0 software package. Qualitative variables are described by absolute (median Me and quartiles Q1–Q3) and relative frequencies (percentages). The Mann–Whitney test was applied to assess the statistical significance of differences between level of HbA1c, plasma glucose level, BMI and arterial hypertension DM2 patient and control groups. The critical level of significance was accepted at *p* < 0.05. Statistical power of the study was 0.8. To compare the frequencies of allele combinations, the χ^2^ criterion was used with the Yates correction for continuity. The association of polymorphisms with DM2 was analyzed by determining the odds ratio criterion (OR) and 95% confidence interval (95% CI).

## 3. Results

It was discovered that the median index of HbA1c in patients with DM2 was 8.45% (7.15–9.95%). The fasting blood glucose level in this group of patients was 9.2 mmol/L (6.1–11.1 mmol/L) and the cholesterol concentration reached 5.1 mmol/ L (4.6–7.3 mmol/L). The median values for blood pressure were systolic, 130.0 mmHg (110–140 mmHg) and diastolic, 85.0 mmHg (80.0–90.0 mmHg). The average BMI was counted at 33.9 kg/m^2^ (26.0–38.7 kg/m^2^) (Table 1). In the control group (Table 1) normoglycaemia, normocholesterolaemia, and physiological levels of HbA1c, as well as normotensia, were detected.

Among patients with DM2, the most common ADIPOQ gene genotype was TT (rs2241766) (50.7%), which was observed less in the control group (87.0%; *p* < 0.001) (Table 2). The number of the ADIPOQ gene’s genotypes TG and GG + 45 T/G (rs2241766) was significantly lower than in the control group (11.0% and 2.0%, respectively) compared to the group of diabetic patients (32.4% and 16.9%, respectively; *p* < 0.001). TG and GG genotypes were associated with the risk of developing DM2 (OR (95% CI) = 3.81 (1.79–8.09) and OR (95% CI) = 10.0 (2.25–44.7). However, both combinations of GG and GT + 276 G/T (rs1501299) (48.9% and 47.8%, respectively) did not differ significantly, compared with the control group (43.0% and 48.0%, respectively). The genotype TT (rs1501299) of the ADIPOQ gene in the DM2 patient group was found to a lesser extent than in the control group (3.3% and 9.0%, respectively; Table 2). 

**Figure 1:**
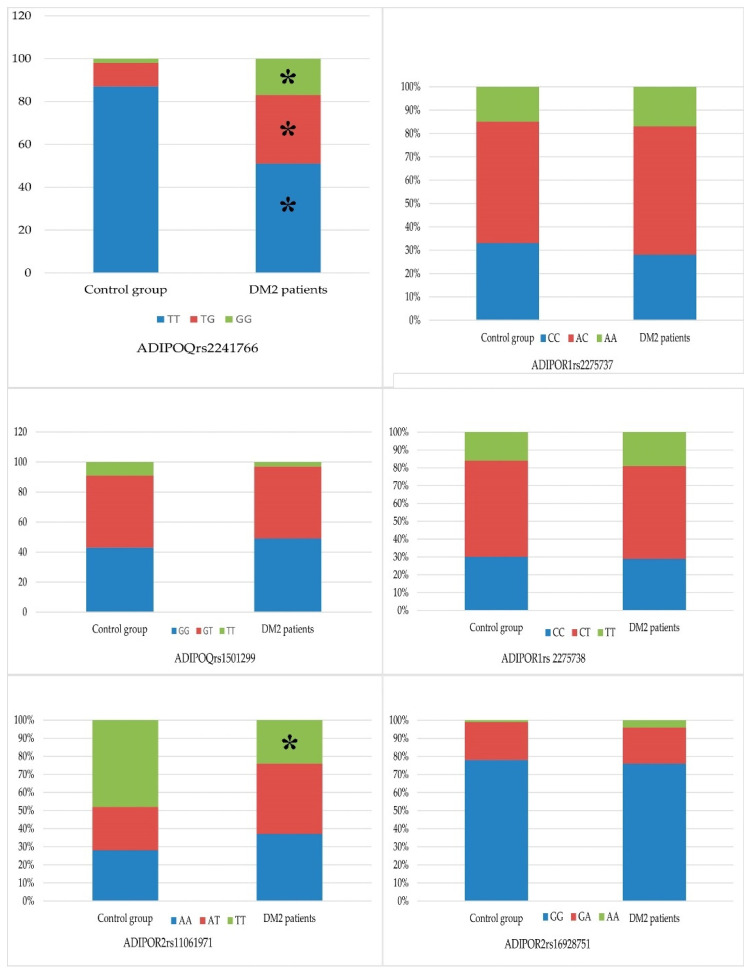
Distribution of genotype polymorphism’s alleles in patients with diabetes mellitus in comparison with healthy individuals. Notes: the symbols of genotypes are given in the graphs, *—*p*-value < 0.05.

Analysis of the frequency of ADIPOR1 allelic variants in DM2 patients demonstrated the most common genotypes to be TG polymorphism – 102 T/G (rs2275737) (55.1%) and CT polymorphism – 106 T/C (rs2275738) (52.2%) (in the controls, 52.0 and 54.0%, respectively) (Figure 1). The TT (rs2275737) and CC (rs2275738) genotypes were found at a lower frequency in the DM2 patient group (28.0% and 28.9%, respectively; Table 1), which was less than that in the control group (33.0% and 30.0%, respectively).

Study of the ADIPOR2 gene + 219 A/T (rs11061971) polymorphism in patients with DM2 demonstrated that the AA and AT genotypes had a frequency of 37.2% and 38.6%, respectively (control, 28.0% and 24.0%; χ^2^ = 3.95, *p* = 0.047) (Figure 1, Table 2). The OR (95% CI) for the ADIPOR2 gene genotype AT + 219 A/T (rs11061971) was 1.94 (1.05–3.5). The OR indicator caused us to regard this polymorphism as a risk factor for the development of DM2. The frequency of the ADIPOR2gene TT + 219 A/T (rs11061971) genotype was statistically significantly lower in the group of patients with DM2 (24.2% vs. 48% in the control group; χ^2^ = 11.5, *p* < 0.001), providing evidence that this polymorphism protects against the risk of DM2 development (OR (95% CI) = 0.34 (0.18–0.62)).

The frequency of the ADIPOR2 gene polymorphism + 795 G/A (rs16928751) polymorphic variants GG and GA was 75.9% and 19.8% in the DM2 patients group, respectively, which was less than in the control group (78.0% and 21.0%; (Figure 1)). The most infrequently observed genotype of ADIPOR2 + 795 G/A (rs16928751) was the polymorphic variant AA, 4.3% in the group of diabetic patients and 0.01% in the control group.

The combination of TT genotype of the ADIPOQ gene + 45 T/G (rs2241766) was found in 24.5% and of the GT genotype—in 17.8% of carriers (Figure 1). The most common combination of ADIPOR1 gene variants was TG − 102 T/G (rs2275737)/CT − 106 T/C (rs2275738), observed in 49.0% of patients with DM2. The next two most common combinations of ADIPOR1 gene variants were TT − 102 T/G(rs2275737)/CC − 106 T/C (rs2275738), observed in 26.5% of patients, and GG − 102 T/G (rs2275737)/TT − 106 T/C (rs2275738), observed in 20.1%; 14.4% of patients were carriers of both the TT genotype of the ADIPOQ gene polymorphism + 45 T/G (rs2241766) and the GG − 102 T/G (rs2275737)/CC − 106 T/C (rs2275738) ADIPOR1 gene polymorphism.

When studying the combinations of ADIPOR2 gene polymorphism + 219 A/T (rs11061971) and + 795 G/T (rs16928751), the most frequent combinations were AT + 219 A/T (rs11061971)/GG + 795 G/T (rs16928751) (31.2%) and AA + 219 A/T (rs11061971)/GG + 795 G/T (rs16928751) (31.1%) (Figure 1). These combinations appeared with equal frequency (17.8%) in DM2 patients who were carriers of the ADIPOQ gene TT genotype + 45 T/G (rs2241766). The frequency of the ADIPOR2 gene combination AT + 219 A/T (rs11061971)/GG + 795 G/T (rs16928751) was detected equally (14.4%) in carriers of the GT and GG ADIPOQ polymorphism + 276 G/T (rs1501299). The frequency of the AA + 219 A/T (rs11061971)/GG + 795 G/T (rs16928751) combination in GT and GG carriers of the ADIPOQ polymorphism + 276 G/T (rs1501299) was 15.5% and 13.3%, respectively.

It was found that carriers of the ADIPOQ gene’s GG genotype + 276 G/T (rs1501299) had the higher level of the blood glucose concentration in 1.16 times than carriers of GT and TT + 276 G/T (rs1501299) (*p* < 0.05) (Table 3). The carriers of the ADIPOR1 gene’s TG − 102 T/G (rs2275737) genotype had the higher level of HbA1c comparative TT − 102 T/G (rs2275737) (in 1.36 times, *p* < 0.05). The carriers of ADIPOR1 gene’s TT − 106 T/C (rs2275738) genotype had the higher concentration of blood glucose in 1.14 times than carriers CC − 106 T/C (rs2275738) (*p* < 0.05) and TT − 106 T/C (rs2275738) in (in 1.1 times, *p* < 0.05).

A relationship was also found between the GA + 795 G/A (rs16928751) genotype of the ADIPOR2 gene and the BMI index. BMI in patients with GA + 795 G/A (rs16928751) genotype was higher in 1.21 times than BMI in GG + 795 G/A (rs16928751) genotype (*p* < 0.05).

## 4. Discussion

The pathogenesis of MS and DM2 predisposition is based on a complex of mechanisms. Adiponectin plays a significant role in glucose metabolism regulation and IR [17]. It has been demonstrated that both levels of adiponectin in the blood serum and the risk of the development of DM2 depend on the polymorphism of the genes [11].

A study carried out in a Han Chinese population of individuals with SNP + 276 of the GG genotype indicated that they had a significantly increased risk of developing DM2 [18]. On the other hand, another study stated that the ADIPOQ gene G rs1501299 polymorphism hardly affected the risk for DM2 in Chinese populations [19]. A Kashmiri population was found to have a non-significant association at SNP + 45, while a statistically significant association of SNP + 276 with the variant genotype (GT + TT) of the ADIPOQ gene was related to the risk of developing DM2 and MS [20]. Moreover, as mentioned above, the genetic polymorphism ADIPOQ rs2241766 (+ 45T > G), genotype GG, was found to correlate with the progression of diabetic nephropathy in an Asian population [19]. Similar findings observed among Italian, French, and Swedish populations did not reveal an association between the SNP + 45 T/G genotype of the adiponectin gene and IR. At the same time, Kaur et al. [21] proved that there was a significant association between SNP + 276 in MS and DM2 in Northern Indian population. It was also displayed that the +276 G/T (rs1501299) polymorphism of the ADIPOQ gene is associated with the risk of obesity in Caucasians [22]. The pathogenesis of nephropathy is tightly bound to glucose toxicity, increased blood pressure, and cholesterol levels, which, as mentioned earlier, were associated with the GG genotype of the ADIPOQ gene (+45T > G rs2241766) [23]. It was also discovered that the GG genotype of the ADIPOQ polymorphism (+45T > G rs2241766) is associated with the phenotypes of hypertension and dyslipidemia in Asian populations [24].

The meta-analyses stated that the allele of variant G rs2241766 was negatively associated with the risk of metabolically controlled obesity; the allele of variant T rs1501299 displayed a decrease in the chances for metabolically controlled obesity and metabolically uncontrolled normal weight in the Chinese population [25]. Additionally, according to the study of Howlader et al. [6], two single-nucleotide polymorphisms of ADIPOQ (rs2241766 and rs1501299) were associated with the risk of dyslipidemia; polymorphism rs1501299 was significantly associated with a susceptibility to ischemic heart disease in East Asia polymorphism rs2241766 was directly related to ischemic heart disease in Europe, East Asia and South Asia [26].

In the Crimean population studied, the most common polymorphic variants of the ADIPOQ gene were TT (+45 T/G, rs2241766) and GT (+276 G/T, rs1501299). In addition, a relationship was found between carriers of the ADIPOQ gene’s GG (rs1501299) genotype and high blood glucose concentrations.

In meta-analysis data from the risk of developing DM2, DM2 was associated with the CC genotype of the ADIPOR1 gene (rs2275737) in a mixed Latin American population [27]. The genotypes AA and GG of the ADIPOR1 gene rs2275738 polymorphism were connected with an increase in the HOMA–IR index and gestational diabetes in the Russian population [28].

In our study, the most common allelic variants of ADIPOR1 in the Crimean patients with DM2 were TG (rs2275737) (*p* < 0.05) and CT (rs2275738). Carriers of the ADIPOR1 gene’s TG (rs2275737) genotype had the highest level of HbA1c (*p* < 0.05), and carriers of the ADIPOR1 gene’s TT (rs2275738) genotype had the most elevated blood glucose concentrations (*p* < 0.05).

The type 2 adiponectin receptor gene, unlike type 1, is expressed mainly by liver cells. This may be of additional importance in the development of glucose tolerance;, since the liver is one of the main places, glucose is stored in the human body. While studies have provided evidence of a relationship between the risk of developing DM2 and the polymorphic marker rs11061971 of the ADIPOR2 gene in the Russian population, no such statistically significant relationships were found for the polymorphic marker rs16928751. Other studies have demonstrated that the polymorphic marker rs11061971 of the ADIPOR2 gene, but not rs16928751, correlates with the risk of DM2 in the Russian population [29].

Allele + 219T rs11061971 and allele + 795A rs16928751 of the ADIPOR2 gene were found to be significantly associated with higher blood pressure in patients with DM2 in the Russian population. In addition, in the same population, for the polymorphic marker rs11061971 of the ADIPOR2 gene, carriers of the A allele had a decreased risk of developing DM2, while carriers of the T allele had an increased risk [15]. The rs16928751 variant was associated with raised fasting triglyceride levels in Europeans with MS [9]. 

In our study, we also found a relationship between the ADIPOR2 gene rs16928751 polymorphism and high BMI index values. The BMI numbers were highest in carriers of the GA genotype compared to other variants of the ADIPOR2 gene’s rs16928751 polymorphism. 

## 5. Conclusions

To sum up, based on the data obtained, we conclude that, in the Russian population, the genes responsible for insulin sensitivity (ADIPOQ, ADIPOR1, and ADIPOR2) are much less associated with the development of DM2 than stated in the data of studies carried out in other population groups.

While the T allele of the rs16928751 polymorphism of the ADIPOR2 gene was associated with an increased risk of DM2 in the Russian population, full genomic searches did not reveal any association of this gene with DM2. 

In addition, a significant difference was found in the gene variants associated with the risk of DM2 in the Crimean population, which helped to establish the relationship between carrying the ADIPOQ gene’s GG (rs1501299) genotype and hyperglycemia. A high BMI index, known to be a key factor in the development of DM2, was found to be strongly associated with the GA genotype of the ADIPOR2 gene’s rs16928751 polymorphism. Additionally, the unique allelic variants of the ADIPOR1 gene related to DM2 were discovered in the Crimean population; the TG rs2275737 genotype was related to high levels of HbA1c and the TT rs2275738 genotype—to hyperglycemia.

The data indicate the importance of studying genetic factors that predispose people to the development of DM2. Recent research has made it clear that the contribution of genetic factors to the development of DM2 varies significantly from population to population. The identification of genetic DM2 risk markers will lead to a better understanding of the main pathological mechanism responsible for its development. In addition, it will aid physicians in choosing the optimal therapy for the disease, as well as helping prevent the development of DM2. To objectify the distribution of risk alleles, further studies with the expansion of the sample volume among DM2 patients and healthy individuals are highly recommended.

### Limitations of the Study

The factors limiting the significance of the results and conclusions of the study include the fact that the research was performed in a specific cohort of the population in the Republic of Crimea. The results obtained can be used for risk assessments of DM2 in other regions only after their confirmation in an independent study.

## Figures and Tables

**Table 1:** Characteristics of the whole study cohort.

	Control Group		DM2 Patients	
	Me	Q1–Q3	Me	Q1–Q3
Age (years)	61	52–70	61	52–70
HbA1c (%)	4.8	4.1–6.0	8.45	7.15–9.95
fasting plasma glucose level, mmol/L	5.2	3.6–5.8	9.2 *	6.1–11.1
Cholesterol (mol/L)	4.6	3.6–6.2	5.1	4.6–7.3
BMI (kg/m^2^)	24.6	21.4–28.9	33.9 *	26.0–38.7
Systolic blood pressure (mmHg)	110	90–118	130.0 *	110–140
Diastolic blood pressure (mmHg)	72	65–80	85.0	80.0–90.0

*—*p*-value < 0.05 as compared to control.

**Table 2:** Adiponectin genes, receptor alleles, and frequency of distribution of genotype polymorphism in patients with diabetes mellitus.

Genes	Polymorphism	Control Group			DM2 Patients			χ^2^, *p*	OR (95% CI)
		Allele Combination	Number of Cases		Allele Combination	Number of Cases			
			Abs.	Proportion					
						Abs.	Proportion		
ADIPOQ	+45 T/G rs2241766	TT	87	0.87	TT	105	0.507	30.3 <0.001	0.1 (0.07–0.31)
		TG	11	0.11	TG	67	0.324	11.8 <0.001	3.81 (1.79–8.09)
		GG	2	0.02	GG	35	0.169	11.4 <0.001	10.0 (2.25–44.7)
	+ 276 G/T rs1501299	GG	43	0.43	GG	101	0.489	ns	
		GT	48	0.48	GT	99	0.478	ns	
		TT	9	0.09	TT	7	0.033	ns	
ADIPOR1	−102 T/G rs2275737	TT	33	0.33	TT	58	0.280	ns	
		TG	52	0.52	TG	114	0.551	ns	
		GG	15	0.15	GG	35	0.169	ns	
	−106 T/C rs 2275738	CC	30	0.30	CC	60	0.289	ns	
		CT	54	0.54	CT	108	0.522	ns	
		TT	16	0.16	TT	39	0.189	ns	
ADIPOR2	+219 A/T rs11061971	AA	28	0.28	AA	77	0.372	ns	
		AT	24	0.24	AT	80	0.386	3.95, 0.047	1.94 (1.05–3.5)
		TT	48	0.48	TT	50	0.242	11.5, <0.001	0.34 (0.18–0.62)
	+795 G/A rs16928751	GG	78	0.78	GG	157	0.759	ns	
		GA	21	0.21	GA	41	0.198	ns	
		AA	1	0.01	AA	9	0.043	ns	

Notes: ns, not significant; OR, odds ratio.

**Table 3:** The components of MS in patients with DM2 with respect to the adiponectin genes and their receptor polymorphism genotypes.

Genotypes		HbA1c (%)	Glucose (mol/L)	Cholesterol (mol/L)	BP Systolic (mmHg)	BP Diastolic (mmHg)	BMI (kg/m^2^)
Control Group		4.8	5.2	4.6	110	72	24.6
		(4.1–6.0)	(3.6–5.8)	(3.6–6.2)	(90–118)	(65–80)	(21.4–28.9)
ADIPOQ							
+45 T/G rs2241766	TT	8.65	9.4	4.9	130	83	33.2
		(6.8–10.2)	(6.3–11.7)	(4.2–7.9)	(110–140)	(80–90)	(26.0–38.7)
	GT	8.6	8.8	5.7	140	85	33.9
		(6.8–10.3)	(6.9–11.0)	(4.8–7.3)	(110–155)	(80–90)	(27.3–40.0)
	GG	8.9	10.4	5.4	135	90	33.0
		(8.4–9.5)	(6.1–12.0)	(4.8–6.7)	(106–150)	(80–101)	(26.7–34.3)
+276 G/T rs1501299	GG	8.5	10.2	5.3	130	85	33.9
		(6.5–9.9)	(6.3–11.6) *	(4.6–7.3)	(110–145)	(80–95)	(29.1–36.3)
	GT	8.6	8.8	5.6	130	90	33.6
		(7.5–10.3)	(6.1–10.7)	(4.8–7.2)	(110–140)	(80–95)	(26.7–41.5)
	TT	9.0	8.4	6.6	130	80	33.9
ADIPOR1							
102 T/G rs2275737	TT	6.6	6.6	5.8	130.0	80	31.9
		(6.1–8.4)	(5.1–9.1)	(4.7–6.8)	(120–150)	(80–85)	(27.8–34.3)
	TG	9.0	9.6	5.6	130	85	34.1
		(7.2–10.5) *	(7.9–11.8)	(4.9–7.3)	(110–140)	(80–90)	(28.8–37.7)
	GG	8.8	9.8	5.1	140	90	36.5
		(8.3–9.9)	(6.9–11.0)	(4.6–6.2)	(116–160)	(87.5–100.5)	(28.4–40.9)
−106 T/C rs2275738	TT	8.7	10.0	5.3	140	90	34.3
		(7.6–9.9)	(6.2–11.3) *	(4.8–6.7)	(105–160)	(80–101)	(26.7–40.0)
	CT	8.3	9.2	5.4	130	85	34.7
		(6.8–10.0)	(7.3–11.2)	(4.4–7.8)	(110–140)	(80–90)	(31.8–41.5)
	CC	9.0	8.8	6.2	130	80	28.3
		(6.2–10.2)	(6.3–10.7)	(4.9–7.0)	(120–160)	(80–100)	(26.0–33.9)
ADIPOR2							
+219 A/T rs11061971	AA	8.8	9.75	5.7	125	87.5	34.3
		(7.6–9.9)	(7.9–11.3)	(4.8–6.7)	(115–140)	(75–101)	(26.7–40.0)
	AT	8.7	9.8	5.2	130	80	31.2
		(6.5–10.5)	(5.8–11.7)	(4.6–6.4)	(130–155)	(80–90)	(26–36.6)
	TT	7.9	8.3	5.4	135	87.5	34.2
		(6.7–9.0)	(6.9–10.0)	(4.9–6.5)	(123–140)	(80–90)	(30.9–40.8)
+795 G/A rs16928751	GG	8.4	8.8	4.9	130	82.5	32
		(6.8–10.1)	(6.1–11.3)	(4.4–7.3)	(110–140)	(70–90)	(26–34.6)
	GA	8.3	7.9	5.9	130	85	38.7
		(6.2–10.5)	(6.2–11.4)	(5.4–10.5)	(110–130)	(80–104)	(33.5–41.5) *
	AA	7.8	9.5	5.5	140	90	30
		(6.7–8.9)	(6.9–12.0)	(4.8–6.2)			

Notes. HbA1c, glycosylated hemoglobin; BP systolic, systolic blood pressure; BP diastolic, diastolic blood pressure; BMI, body mass index. * *p*, the critical level of significance was accepted at *p* < 0.05.

## Data Availability

Data are available upon request from the author (I.S.S.).
